# Riemannian Geometry for Noise-Robust Covariance Network Analysis of Schizophrenia EEG: Geometric-Entropic Signatures of Dysconnectivity

**DOI:** 10.3390/e28060644

**Published:** 2026-06-08

**Authors:** Rui Song, Jinhan He, Jun Wang

**Affiliations:** 1 Smart Health Big Data Analysis and Location Services Engineering Research Center of Jiangsu Province, School of Chemistry and Life Sciences, Nanjing University of Posts and Telecommunications, Nanjing 210023, China; b23100307@njupt.edu.cn (R.S.); b23100323@njupt.edu.cn (J.H.); 2 Smart Health Big Data Analysis and Location Services Engineering Research Center of Jiangsu Province, School of Applied Technology, Nanjing University of Posts and Telecommunications, Nanjing 210023, China

**Keywords:** Riemannian geometry, EEG, schizophrenia, covariance manifold, information geometry, entropy, brain connectivity, affine-invariant metric, nonlinear coupling

## Abstract

Functional brain networks in schizophrenia (SZ) are often characterized by covariance-based measures, yet covariance matrices live on a curved geometric structure rather than in ordinary Euclidean space, complicating noise-robust inference from scalp EEG. We develop a Riemannian Geometry-based Adaptive Nonlinear Coupling Analysis (RGA-NCA) framework that integrates the affine-invariant Riemannian metric (AIRM), tangent space mapping (TSM), and an anatomically adaptive artifact rejection (AAAR) strategy accounting for regional signal-to-noise heterogeneity. The framework is grounded in the observation that Euclidean summaries of symmetric positive definite matrices are sensitive to noise-driven volume inflation, whereas geodesic distances on the manifold emphasize shape deformation. RGA-NCA was evaluated on four benchmark dynamical systems, a supplementary multichannel EEG-like sample covariance simulation, and a public button-tone SZ/HC EEG dataset associated with the auditory feedback paradigm described by Ford et al. (81 subjects; 49 SZ, 32 healthy controls). Compared with Euclidean and linear baselines, RGA-NCA showed lower sensitivity to noise-driven distance distortion and yielded clearer group-level contrasts in the tested ROI analyses; all four pre-specified frontotemporal and parietal channel pairs remained significant after Benjamini–Hochberg FDR correction. The resulting patterns are consistent with reduced long-range connectivity together with localized hyper-synchronization-like effects in SZ. Quantitatively, the Riemannian structural sensitivity index $\left(\mathrm{sim}=\exp\left(-d^{2}/4\right)\right)$ remained high across all tested SNR levels (−20 to +10 dB; 50 Monte Carlo trials per level; range 0.936–0.964), with only a 0.026 endpoint change between +10 and −20 dB, whereas the Euclidean metric fell from 0.922 at +10 dB to 0.000 at −20 dB. These findings support Riemannian modeling as a candidate strategy for noisy covariance-based neural data, pending validation in larger independent cohorts.

## 1. Introduction

Schizophrenia (SZ) is a severe chronic psychiatric disorder increasingly framed as a disorder of large-scale brain connectivity, or “dysconnectivity syndrome” [1]. In this paper, SZ denotes schizophrenia patients and HC denotes healthy controls. EEG is a natural tool for probing these fast neurodynamical abnormalities because of its millisecond-scale temporal resolution, yet extracting reliable nonlinear topological features from scalp signals remains challenging in practice [2].

Early studies relied on linear metrics such as coherence and Pearson correlation [2,3]. However, neural signals exhibit nonlinear and non-stationary dynamics, and linear summaries may miss phase coupling or amplitude modulation between neuronal populations. Analyses of nonlinear EEG dynamics and information-geometric representations have therefore become central to brain network modeling [4,5]. To overcome this limitation, kernel Granger causality analysis in reproducing kernel Hilbert spaces (RKHS) [6,7] attempts to capture high-order nonlinear interactions via the kernel trick. However, many kernel implementations assume a Euclidean feature space and apply Euclidean distance directly to covariance-derived features.

**Figure 1:**
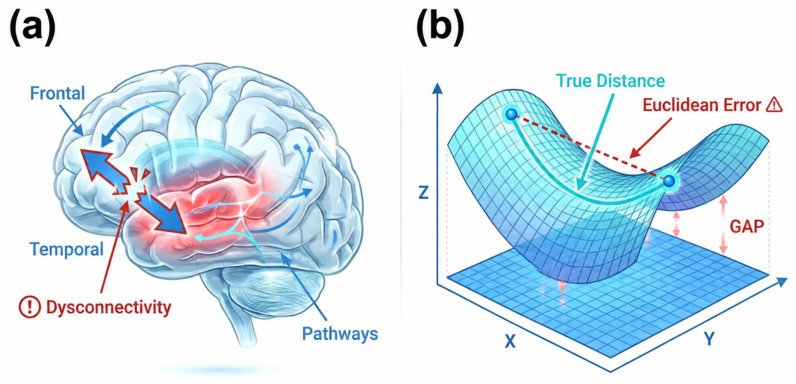
Conceptual illustration. (**a**) Neurophysiological context: in a healthy brain, the frontal lobe suppresses the temporal auditory cortex via an efference copy, and disruption of this connection in SZ patients has been associated with auditory hallucinations [12,13,14]. (**b**) Geometric mismatch: on a non-Euclidean Riemannian manifold, the geodesic distance along the surface (teal, “True Distance”) differs from the straight-line Euclidean distance (red dashed, “Euclidean Error”); the gap between the straight chord and the curved surface illustrates how Euclidean summaries depart from the manifold and distort statistical comparisons.

A central issue, illustrated in Figure 1, is this “geometric mismatch”: EEG covariance matrices live on the manifold of symmetric positive definite (SPD) matrices rather than in a flat Euclidean space [5,8,9,10]. From an information-geometric perspective [5], manifold distances can be interpreted in terms of distributional distinguishability rather than ordinary Euclidean displacement. Our theoretical analysis and simulations indicate that this mismatch increases the sensitivity of Euclidean summaries to non-stationary covariance perturbations, obscuring structured connectivity differences. Standard preprocessing pipelines compound the problem by treating all regions uniformly, even though signal-to-noise ratio (SNR) varies substantially across the scalp; frontal channels are more vulnerable to ocular artifacts, whereas occipital signals are typically cleaner [2,11]. This regional heterogeneity makes the denoising bias–variance trade-off difficult to manage, and motivates the anatomically adaptive artifact rejection introduced in Section 3.

**Figure 2:**
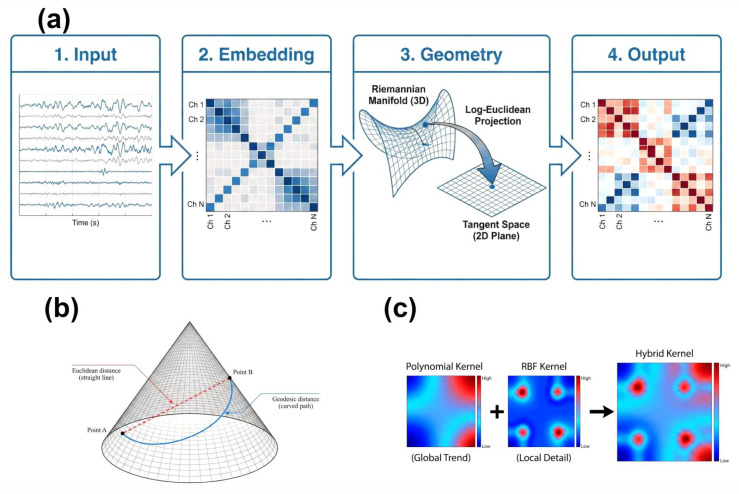
Overview of the RGA-NCA workflow. (**a**) Broadband processing flow from raw signals to Riemannian manifold construction. (**b**) Geometric principle of tangent space projection (log-map $\log_{P}\left(\;\cdot \;\right)$). (**c**) Weight allocation of the adaptive hybrid kernel in tangent space. The layout highlights the correspondence between the overall processing pipeline and its local geometric components.

To address these challenges, we propose the Riemannian Geometry-based Adaptive Nonlinear Coupling Analysis (RGA-NCA) framework (Figure 2). RGA-NCA is positioned as a complementary geometry-aware pipeline rather than a replacement for prior Riemannian EEG approaches, and it emphasizes (i) covariance-manifold distance modeling with AIRM and tangent space projection, (ii) an anatomically adaptive artifact rejection (AAAR) strategy tailored to regional SNR heterogeneity, and (iii) an auxiliary hybrid kernel similarity descriptor for multi-scale nonlinear coupling analysis. Whereas many prior Riemannian EEG studies focus on classification performance, our emphasis is on comparative noise sensitivity, geometric interpretability, and group-level connectivity characterization in schizophrenia.

The remainder of the paper is organized as follows. Section 2 reviews traditional kernel-based coupling methods and the geometric assumptions that limit them on non-stationary EEG covariance data. Section 3 develops the RGA-NCA framework and details the algorithm. Section 4 reports the experimental results. Section 5 discusses the findings and their limitations, followed by the conclusion.

## 2. Background and Related Work

Before introducing the proposed framework, we review the kernel-based coupling methods that motivate it and highlight the geometric assumptions that limit them on non-stationary EEG covariance data. Because EEG signals show nonlinearity, non-stationarity, and anatomical SNR heterogeneity, traditional linear analyses may miss group differences linked to high-order interactions; kernel methods were introduced to recover such interactions, but most implementations inherit a Euclidean feature-space assumption that we revisit below.

Nonlinear Granger causality analysis uses the kernel trick to map low-dimensional time series into a high-dimensional, possibly infinite-dimensional, RKHS [7]. In this feature space, complex nonlinear dependencies can be recast as linear regression problems.

Let two time series be X and Y. The Granger causality index (GCI) is defined as the logarithmic ratio of prediction error variances:

(1) $$GCI_{Y\to X}=\ln\frac{\mathrm{var}(E_{X})}{\mathrm{var}(E_{X|Y})}$$

where $E_{X}$ is the residual when predicting using only the history of X, and $E_{X|Y}$ is the residual when predicting using the history of both X and Y. A positive value of $GCI_{Y\to X}$ indicates that the past state of Y carries additional predictive information for X.

### 2.1. Sigmoid Kernel: Neural Activation Analog

The sigmoid kernel originates in artificial neural networks [15,16], particularly multi-layer perceptrons (MLPs), and is defined as

(2) $$K_{\mathrm{Sig}}\left(x,y\right)=\tanh\left(\alpha x^{⊤}y+c\right)$$

where α is a scaling factor and *c* is the intercept. The hyperbolic tangent emulates nonlinear saturation responses and provides a flexible nonlinear mapping.

In brain network causality analysis, however, the sigmoid kernel faces a fundamental mathematical issue: it is conditionally positive definite rather than a Mercer kernel [17]. For certain (α,c), the Gram matrix can have negative eigenvalues, violating Mercer’s theorem. This non-semi-positive definiteness causes the induced “distance” in feature space to lose its geometric meaning.

### 2.2. Gaussian Kernel (RBF): Local Approximation

The Gaussian kernel, or radial basis function (RBF), is the most widely used local stationary kernel [15]:

(3) $$K_{\mathrm{RBF}}\left(x,y\right)=\exp\left(-\frac{{∥x-y∥}^{2}}{2\sigma^{2}}\right)$$

where σ is the bandwidth controlling the local scope. The Gaussian kernel measures similarity through Euclidean distance and maps data to an infinite-dimensional feature space.

Although the Gaussian kernel performs well in interpolation, it has a geometric limitation in EEG covariance analysis. It assumes that data are embedded in a flat Euclidean space, with similarity determined entirely by ${∥x-y∥}^{2}$. However, EEG sample covariance matrices (SPD matrices) form a non-Euclidean Riemannian manifold under affine-invariant geometry, so applying Euclidean distance inside the exponential disregards the manifold’s intrinsic curvature [17].

### 2.3. Polynomial Kernel: Global High-Order Interaction

The polynomial kernel is a global kernel designed to capture high-order couplings between signals through explicit feature mapping [18]:

(4) $$K_{\mathrm{Poly}}\left(x,y\right)={\left(x^{⊤}y+c\right)}^{d}$$

where *d* is the polynomial degree. The main drawback is non-locality and sensitivity to amplitude. In the low-SNR environment of scalp EEG, particularly with EOG and EMG artifacts common in schizophrenia recordings, artifacts manifest as high-amplitude outliers. Lacking the local decay of the Gaussian kernel, the polynomial kernel can amplify these outliers [17].

## 3. Methods

This section introduces the mathematical framework used to quantify nonlinear topological differences in schizophrenia brain networks. An overview of the complete RGA-NCA workflow is provided in Figure 2; the following subsections detail each component. To address the geometric mismatch and SNR heterogeneity faced by the traditional kernel methods reviewed in Section 2 on non-stationary EEG signals, we propose the Riemannian Geometry-based Adaptive Nonlinear Coupling Analysis (RGA-NCA), which integrates neuroanatomical priors with information geometry. The framework couples covariance-manifold distance modeling, anatomically adaptive preprocessing, and an auxiliary hybrid kernel descriptor, and we evaluate it along three axes: geometric fidelity, noise sensitivity, and group-contrast sensitivity.

### 3.1. Geometrical Framework: From Euclidean to Riemannian

The quantification of brain network topology depends on the geometric structure of the underlying state space [5]. Here, we treat EEG sample covariance matrices (SCMs) not merely as statistical estimators but as points on a curved differentiable manifold.

**SPD manifold and curvature:** Let $S_{N}^{++}$ denote the space of N×N SPD matrices [8,9]. Under the affine-invariant geometry, this manifold is not a vector space; the shortest path between two points is a geodesic on the manifold, not a straight line in ambient Euclidean coordinates.

**Figure 3:**
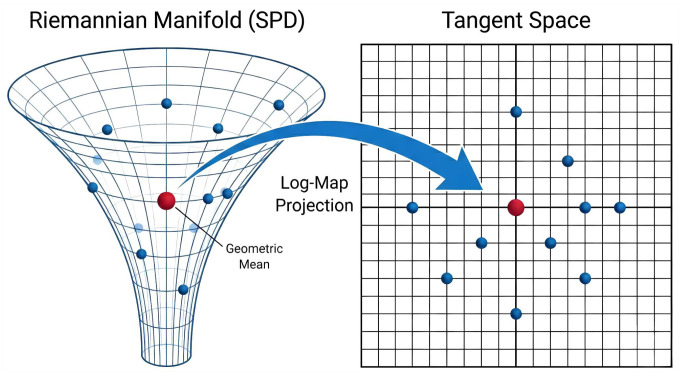
Riemannian geometric mean and tangent space projection mitigate covariance volume inflation. EEG covariance descriptors are symmetric positive definite (SPD) and lie on a curved manifold (left, depicted as the SPD cone). The reference mean used by RGA-NCA is the Riemannian (Fréchet) geometric mean, depicted as the larger red sphere in both panels. Sample covariance matrices are shown as smaller blue spheres. The geometric mean lies on the manifold along the geodesics connecting these sample points. By contrast, a Euclidean arithmetic mean would leave the manifold surface and increase the matrix determinant (volume inflation), amplifying noise-related variance. Points are then carried to the Euclidean tangent space at this reference through the logarithmic map (Log-Map projection, right), where the affine-invariant geodesic relationships are locally linearized for efficient computation.

**Geometry of covariance inflation:** The noise-related covariance inflation studied here arises from applying a Euclidean metric (Frobenius norm) to matrices that lie on a curved SPD manifold. Under affine-invariant SPD geometry, geodesic and Euclidean volume–distance relationships differ substantially (Figure 3). Consequently, Euclidean distances applied to noisy covariance matrices can over-weight small covariance perturbations. From an information-theoretic standpoint, this corresponds to increased differential entropy of the noise distribution in flat-space embeddings; the Riemannian metric constrains distances to the manifold’s intrinsic geometry and reduces this entropy inflation.

To address this, we equip $S_{N}^{++}$ with the AIRM [19]. The geodesic distance $\delta_{R}$ between two points $P_{1}$ and $P_{2}$ is

(5) $$\delta_{R}\left(P_{1},P_{2}\right)={∥\log\left(P_{1}^{-1/2}P_{2}P_{1}^{-1/2}\right)∥}_{F}={\left(\sum_{i=1}^{N}\ln^{2}\lambda_{i}\right)}^{1/2}$$

where ${∥\;\cdot \;∥}_{F}$ is the Frobenius norm, log(·) is the matrix logarithm, and $\lambda_{i}$ are the generalized eigenvalues of $P_{1}^{-1}P_{2}$. This metric is invariant to affine transformations such as the linear scaling induced by electrode impedance changes.

### 3.2. Tangent Space Projection and Linearization

To enable efficient computation while preserving geometric structure, we adopt a local linearization strategy based on TSM [9]. For any reference point $P_{ref}$ on the manifold $S_{N}^{++}$, the tangent space $T_{P_{ref}}S_{N}^{++}$ is a Euclidean vector space tangent to the manifold at that point.

**Reference point and logarithmic mapping:** The tangency reference $P_{ref}$ is the Riemannian Fréchet (geometric) mean [20]:

(6) $$P_{ref}=\arg\min_{P\in S_{N}^{++}}\sum_{i=1}^{K}\delta_{R}^{2}\left(P,P_{i}\right)$$

Each manifold point $P_{i}$ is then projected to a symmetric matrix $S_{i}$ in the tangent space via the log-map:

(7) $$S_{i}={\mathrm{Log}}_{P_{ref}}\left(P_{i}\right)=P_{ref}^{1/2}\log\left(P_{ref}^{-1/2}P_{i}P_{ref}^{-1/2}\right)P_{ref}^{1/2}$$

**Vectorization:** Applying the half-vectorization operator to $S_{i}$ yields the feature vector $v_{i}\in R^{M}$ [9].

### 3.3. Adaptive Hybrid Kernel Embedding

The final step of RGA-NCA constructs an adaptive hybrid kernel as a weighted combination of the Gaussian and polynomial kernels:

(8) $$K_{hybrid}\left(v_{i},v_{j}\right)=\lambda \;\cdot \;K_{RBF}\left(v_{i},v_{j}\right)+\left(1-\lambda \right)\;\cdot \;K_{Poly}\left(v_{i},v_{j}\right)$$

where $v_{i},v_{j}$ are tangent space feature vectors and λ∈[0,1] is the mixing factor. Adjusting λ tunes the relative contribution of local RBF-like smoothing and global polynomial interaction structure within the broadband range (1–45 Hz).

For each retained segment, the implementation computes two related but distinct quantities. First, the hybrid kernel value is retained as an auxiliary nonlinear similarity score. For segment *s* and a left-/right-channel pair, $K_{hybrid}\left(v_{L,s},v_{R,s}\right)$ quantifies the tangent space similarity between the two channel-specific SCM descriptors; larger values indicate stronger nonlinear covariance-coupling similarity rather than greater distance. Segment-level kernel scores are summarized as

(9) $${\bar{K}}_{\mathrm{ROI}}=\frac{1}{\left|S\right|}\sum_{s\in S}K_{hybrid}\left(v_{L,s},v_{R,s}\right),$$

providing a subject-level auxiliary kernel-coupling profile for multi-scale nonlinear coupling characterization and kernel-mixture sensitivity analysis. This auxiliary similarity score is not the scalar reported as the primary group statistic in Section 4. Second, the primary subject-level statistic for group inference is the AIRM distance between the two channel-specific SCMs:

(10) $$D_{\mathrm{ROI}}=\frac{1}{\left|S\right|}\sum_{s\in S}\delta_{R}\left(C_{L,s},C_{R,s}\right),$$

where S is the set of retained within-subject segments, and $C_{L,s}$ and $C_{R,s}$ are the left- and right-channel SCMs for segment *s*. Thus, a larger reported Riemannian distance indicates greater covariance-structure divergence between the two channels within a given subject. The group-level comparisons reported in Section 4 use this subject-level Riemannian distance statistic.

### 3.4. EEG Dataset and Preprocessing

The empirical analysis used a public button-tone SZ/HC EEG dataset distributed via Kaggle and associated with the auditory feedback paradigm described by Ford et al. [21]. The analyzed Kaggle cohort comprised 81 subjects (49 SZ and 32 HC). The paradigm was an event-related self-generated auditory feedback task rather than resting-state EEG. In the button-to-tone condition, button presses triggered a 1000 Hz, 80 dB SPL tone of 100 ms duration; yoked passive-listening and button-only control conditions were also included.

Following the dataset documentation and our local preprocessing workflow, EEG was recorded with a 64-channel BioSemi ActiveTwo system at 1024 Hz and resampled to 1000 Hz for downstream analysis. The scalp electrodes followed the extended international 10–20 system and were supplemented by external EOG and mastoid channels during acquisition. Preprocessing included 0.1 Hz high-pass filtering, bad-channel interpolation, canonical correlation analysis (CCA)/independent component analysis (ICA)-based artifact attenuation, common-average rereferencing, and baseline correction using the −100 ms to 0 ms pre-stimulus interval. For RGA-NCA, broadband 1 Hz to 45 Hz signals were analyzed using sliding windows of 500 ms (500 samples) with 250 ms overlap before covariance estimation.

### 3.5. Anatomically Adaptive Artifact Rejection (AAAR)

**Theoretical basis (Bayesian priors and the bias–variance trade-off):** Throughout the AAAR procedure, SD denotes the standard deviation computed after z-scoring the detrended channel segment. AAAR is built on an SNR equilibrium principle:

- **Frontal heavy-tailed prior:** Prefrontal and temporal regions (e.g., F3, F4, T7, T8) are more susceptible to heavy-tailed noise from EOG and EMG due to their proximity to the eyes and facial muscles. In these regions, we apply stricter truncation thresholds (SD≈2.5–3.0) to prioritize removal of high-amplitude outliers. This reduces artifact effects on local manifold geometry and limits ill-conditioned drift of eigenvalues [11,22,23].
- **Parietal/occipital Gaussian prior:** Parietal and occipital regions (e.g., P7, P8, O1, O2) typically show prominent alpha-band activity (8–13 Hz) and higher signal quality. Here we apply looser thresholds (SD≈4.0–5.0) to preserve physiologically meaningful oscillatory phase information and weak high-frequency nonlinear features, reducing the risk of over-denoising clinically relevant structure [11,22,23].

**Figure 4:**
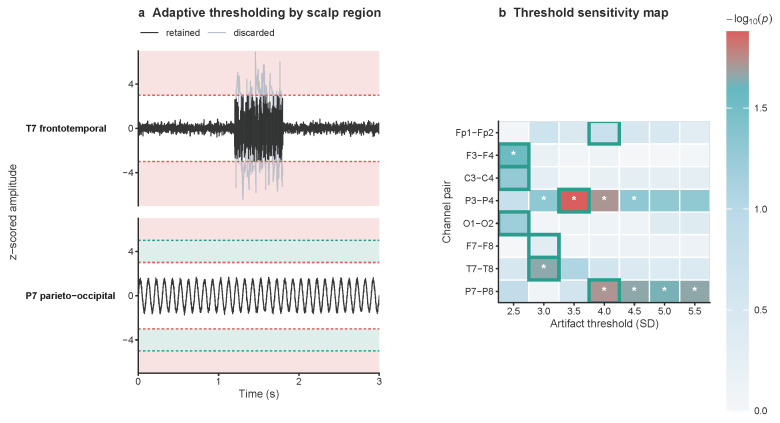
Empirical basis of anatomically adaptive artifact rejection (AAAR). (**a**) A strict threshold (SD=3) attenuates high-amplitude artifacts in frontotemporal regions (T7, top panel), whereas the adaptive threshold preserves alpha-band structure in parieto-occipital regions (P7, bottom panel). (**b**) Heatmap of region-dependent sensitivity to threshold selection ($-\log_{10}P$): the teal outlines indicate the selected adaptive thresholds for each channel pair; asterisks mark cells with p<0.05. SD: standard deviation.

The empirical basis for these region-specific thresholds is illustrated in Figure 4: panel (a) contrasts the effect of a strict threshold on frontotemporal channels with that of an adaptive threshold on parieto-occipital channels, while panel (b) maps the region-dependent sensitivity ($-\log_{10}P$) to threshold selection across all channel pairs.

### 3.6. Statistical Analysis

All group comparisons were two-sided. For demographic variables, age and education were compared with Welch independent-samples *t*-tests, and sex distribution was compared with Fisher’s exact test. For the pre-specified region-of-interest (ROI) analyses, subject-level RGA-NCA distance statistics were first screened with the default robust outlier procedure in MATLAB (MATLAB_R2025b) rmoutliers, applied separately within each group and channel pair. Retained HC and SZ values were then compared with the default two-sample Student *t*-test (MATLAB ttest2). Welch tests were computed as sensitivity checks and are reported in the Supplementary Materials. The four pre-specified ROI tests were corrected with the Benjamini–Hochberg (BH) false discovery rate (FDR) procedure [24] (a step-up correction that controls the expected proportion of false discoveries among rejected hypotheses) at α=0.05. Both raw and BH-adjusted *p*-values are reported; identical adjusted values can arise from the monotonicity constraint of the BH step-up procedure. Exploratory individual-level classification was evaluated separately using leave-one-subject-out cross-validation and was not used as primary evidence for the group-level findings.

## 4. Results

### 4.1. Controlled Dynamical-System Stress Test on SPD Manifolds

Before turning to the neurophysiological dataset, we stress-tested the SPD-manifold operations in a controlled dynamical setting. This analysis was not designed to reproduce multichannel EEG covariance matrices. Instead, it probed the method’s operating range and dynamical fidelity within a **phase-space reconstruction** validation framework, covering input construction, geometric noise resistance, and topological capture under known nonlinear dynamics.

#### 4.1.1. Input Principle: From Scalar Series to Riemannian Manifold

To keep the benchmark physically meaningful, we avoided arbitrarily generated random positive-definite matrices. Instead, we used a **dynamical embedding** strategy that converts one-dimensional time series into geometric objects on the manifold:

- **Trajectory generation:** Scalar time series x(t) were generated for four standard chaotic systems using numerical integration (e.g., fourth-order Runge–Kutta).
- **Phase-space reconstruction:** Following Takens’ embedding theorem, each 1D series x(t) was mapped into an *m*-dimensional pseudo-phase space. A trajectory matrix $X_{i}=\left[x\left(i\right),x\left(i+\tau \right),\ldots ,x\left(i+\left(m-1\right)\tau \right)\right]$ was constructed via sliding windows with embedding delay τ=1 sample and embedding dimension m=4, yielding a 4×500 trajectory matrix per analysis window. This approximates the local attractor geometry in high-dimensional space.
- **Manifold mapping and regularization:** The sample covariance matrix P=Cov(X) captures the second-order ellipsoidal approximation of the attractor in local phase space. To avoid numerical singularities from trajectory contraction and to ensure each matrix lies strictly inside the SPD manifold, we applied diagonal loading regularization ($\epsilon =10^{-3}$) and trace normalization:
  (11) $$P_{input}=\frac{P+\epsilon I}{\mathrm{Tr}(P+\epsilon I)}$$
  Each $P_{input}$ is thereby placed at a well-defined point on the Riemannian manifold $S_{m}^{++}$ associated with a specific dynamical state.

These benchmark covariance matrices are not intended to be generative replicas of EEG channel covariance matrices. In the empirical EEG analysis, each selected channel pair is represented by two delay-embedded channel trajectories, and the Riemannian distance or hybrid kernel statistic is computed between the two channel-specific SCMs. In the benchmark analysis, by contrast, the covariance of a delay-embedded scalar trajectory is used only to create controlled SPD descriptors whose underlying dynamics are known. This design isolates the behavior of the metric under known topology and injected noise while keeping the same SPD-manifold operations used in the real-data pipeline.

#### 4.1.2. Noise Perturbation Analysis

**Figure 5:**
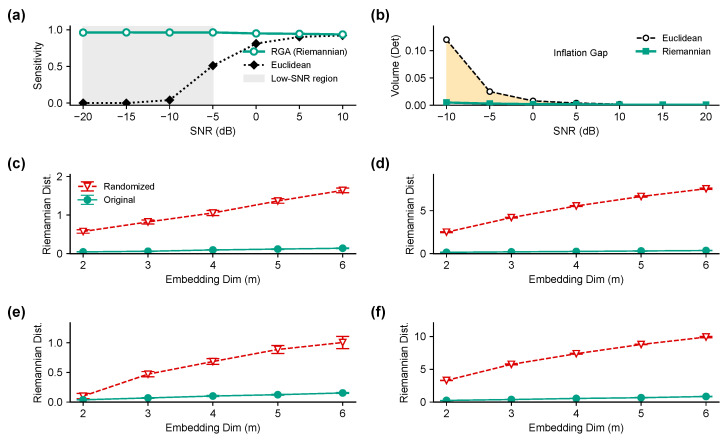
**Multi-dimensional benchmarking of the RGA-NCA framework.** (**a**) Noise robustness. (**b**) Noise-related inflation. (**c**–**f**) Topology reconstruction for four benchmark dynamical systems: (**c**) Hénon map, (**d**) Lorenz system, (**e**) Ikeda map, (**f**) driven Duffing oscillator. Error bars: SD (N=10).

Figure 5 presents the multi-dimensional benchmarking results. The structural sensitivity index used in panel (a) is defined as $\mathrm{sim}=\exp(-d^{2}/4)$, where *d* is the AIRM (Riemannian) or Euclidean Frobenius distance between the noisy and reference covariance matrices; a value closer to 1.0 indicates greater preservation of geometric structure under noise. In panel (a), the Riemannian RGA-NCA sensitivity (teal, open circles) remains high and nearly flat across the tested SNR range (−20 to +10 dB), whereas the Euclidean sensitivity (black dotted, filled diamonds) collapses as SNR decreases; the shaded band marks the low-SNR region (−20 to −5 dB) where the two metrics diverge most. Panel (b) shows that the Euclidean volume increases with noise, whereas the Riemannian metric exhibits comparatively lower volume-related drift. Panels (c–f) report the Riemannian structural distance between original and phase-randomized surrogate data for four dynamical systems spanning discrete maps, continuous flows, delay-feedback dynamics, and externally driven oscillations.

We then examined how the Riemannian metric behaves under non-stationary noise. Injecting additive noise $P_{noise}=P_{true}+\epsilon RR^{T}$ into the benchmark matrix revealed two distinct geometric response patterns (Figure 5a,b):

**Noise-related inflation in Euclidean space:** The Euclidean (Frobenius) metric showed approximately linear sensitivity to noise energy. As SNR decreased, background noise increased the system’s differential entropy, which appeared in Euclidean summaries as increased matrix volume (determinant). This entropy-related inflation can reduce separation between structured dynamics and random perturbations.

**Riemannian response to noise perturbation:** By contrast, RGA-NCA uses the non-Euclidean geometry of the SPD manifold. Because the AIRM emphasizes shape deformation of the covariance ellipsoid rather than its volumetric size, it is less sensitive to the isotropic inflation produced by additive noise. The index remained high even under the strongest tested perturbation, supporting its utility as a noise-resistant geometric descriptor under the tested conditions. Quantitatively, across 50 Monte Carlo trials per SNR level, the Riemannian sensitivity remained in a narrow range (0.936–0.964), with only a 0.026 endpoint change between +10 and −20 dB. Under the same perturbation setting, the Euclidean metric fell from 0.922 at +10 dB to 0.000 at −20 dB, a decrease of 0.922 (Figure 5a).

#### 4.1.3. Validation on Benchmark Dynamical Systems

We selected these systems because they represent qualitatively distinct dynamical regimes encountered in nonlinear time-series analysis. To test the adaptability of this input principle to different types of dynamical attractors, we selected four representative systems (Figure 5c–f), spanning low-dimensional discrete maps, continuous flows, delay-feedback dynamics, and externally driven oscillations.

(1) Discrete Fractal Topology (Henon Map) [25]

As a benchmark for discrete dynamical systems, the Henon map exhibits typical stretching and folding mechanisms, with an attractor possessing a fractal dimension (approx. 1.26):

(12) $$x_{n+1}=1-1.4x_{n}^{2}+y_{n},\;y_{n+1}=0.3x_{n}$$

RGA-NCA reflected nonlinear correlations in its phase space. As the embedding dimension *m* increased, the Riemannian distance between the original attractor and the phase-randomized surrogate data (Figure 5c–f) widened, suggesting that the algorithm is sensitive to fractal-related geometric differences in discrete sequences.

(2) Continuous Manifold Flow (Lorenz System) [26]

To simulate continuous trajectories on neural manifolds, we employed the Lorenz attractor (s=10,r=28,b=8/3). Its “butterfly” structure was reflected as specific covariance pattern transitions in the manifold space. The framework tracked the intrinsic geometric structure of continuous flows with low intra-class variance in this benchmark, supporting its applicability in continuous-time systems.

(3) Infinite-Dimensional Delay Chaos (Ikeda Map)

Neural systems are replete with axonal conduction delays. To validate the algorithm’s ability to handle time-delay feedback, we used the **Ikeda Map**, a physical model describing the dynamics of a light beam in a ring cavity within a nonlinear optical medium [27]:

(13) $$\begin{array}{cc} x_{n+1} & =1+\mu (x_{n}\cos t_{n}-y_{n}\sin t_{n})\\ y_{n+1} & =\mu (x_{n}\sin t_{n}+y_{n}\cos t_{n}) \end{array}$$

where $t_{n}=0.4-6/\left(1+x_{n}^{2}+y_{n}^{2}\right)$ and μ=0.9. Because of the delay, the phase space of this system is theoretically infinite-dimensional. The RGA-NCA-based input principle remained numerically stable when processing such high-dimensional folded systems, reducing singularity-related numerical instability in delay-system benchmarks.

(4) Non-Autonomous Driven Dynamics (Duffing Oscillator)

Finally, to simulate the brain’s response under external sensory stimulation (e.g., auditory evoked potentials), we simulated the driven Duffing oscillator [28]:

(14) $$\ddot{x}+\delta \dot{x}-x+x^{3}=\gamma \cos\left(\omega t\right)$$

Parameters were set to damping δ=0.2, driving force γ=0.3, and angular frequency ω=1.0. Clear separation between original and surrogate trajectories indicated that RGA-NCA could preserve the intrinsic topological signature of the driven system under background fluctuations, which is relevant for analyzing task-based EEG data.

#### 4.1.4. Statistical Significance Testing

To establish statistical decision boundaries, we introduced **Surrogate Data Testing**. For all the above systems, we generated 1000 sets of surrogate data that preserved the amplitude spectrum but destroyed nonlinear phases (Null Hypothesis $H_{0}$: signal is generated by a linear stochastic process). The results showed that the Riemannian coupling indices for all chaotic systems lay outside the 99th percentile of the null distribution (Z-score>4.2), providing evidence against the linear stochastic null model.

#### 4.1.5. Supplementary Multichannel EEG-like SCM Check

Because the controlled dynamical-system benchmark uses delay-embedded scalar trajectories rather than direct channel-by-time EEG matrices, we added a supplementary multichannel EEG-like covariance simulation using the same sample covariance form as the empirical analysis: $X\in R^{64\times 500}$ and $C=\left(X-\bar{X}\right){\left(X-\bar{X}\right)}^{⊤}/\left(T-1\right)$. The simulation used the same HC/SZ sample sizes as the empirical cohort (32/49 subjects), heterogeneous frontotemporal sensor noise, and four implanted channel-wise covariance perturbations matching the pre-specified ROI structure (Supplementary Simulation S1; Figure S1; Table S7). Direct ROI-pair recovery was high in both representations, confirming that the implanted channel-pair perturbations were recoverable in this channel-wise SCM simulation. The main advantage of AIRM appeared in whole-SCM contrast recovery, where it better preserved the global HC–SZ covariance contrast under moderate noise: contrast cosine similarity was 0.862±0.005 versus 0.427±0.043 at +20 dB and 0.800±0.005 versus 0.405±0.034 at +10 dB. A sensitivity grid across implanted-effect scales (0.50×–1.25×) showed that the AIRM advantage in full-SCM contrast recovery remained positive at +20, +10, and 0 dB, but vanished under severe noise (−10 dB). This supplementary analysis is therefore used as a covariance-form consistency check rather than as a generative model of schizophrenia EEG.

### 4.2. Real-World EEG Analysis

#### 4.2.1. Demographics

The analyzed cohort comprised 81 subjects (SZ: 49, HC: 32), with detailed demographic statistics shown in Table 1. As described in Section 3, the data come from an event-related button-tone paradigm rather than a resting-state EEG recording.

#### 4.2.2. Illustrative Geometric Correction

**Figure 6:**
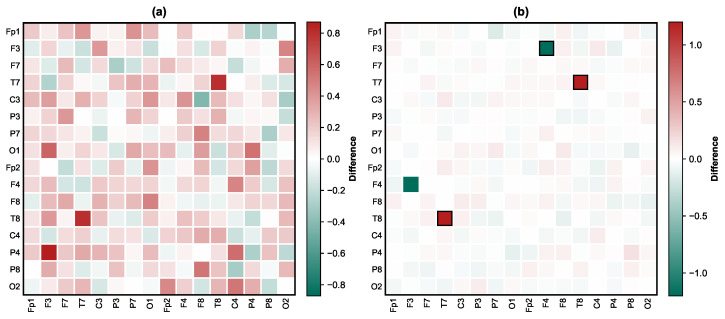
Illustrative geometric correction from Euclidean to Riemannian representation. In both panels, the heatmaps display group-difference statistics derived from subject-level distance statistics, not raw covariance matrices. (**a**) Euclidean distance metric (Frobenius norm applied to sample covariance matrices). The difference matrix shows a diffuse global pattern, with structured connectivity differences less visually separable from inflated background noise. (**b**) Riemannian tangent space distance (AIRM geodesic). After reducing inflation using the geometry-aware pipeline, the map shows a more localized pattern, and sparse connectivity differences in frontotemporal and parietal regions become more apparent.

As shown in Figure 6, the mapping from Euclidean space to the Riemannian tangent space produced a visually more focused contrast pattern in this dataset. Under the Euclidean metric (Figure 6a), the brain network difference matrix showed a diffuse distribution of weak connections. In contrast, the geometry-aware representation (Figure 6b) yielded a sparser pattern in which frontotemporal and parietal differences were more visually apparent. This figure should be interpreted as an illustrative visualization of the geometric correction rather than as independent quantitative proof of a specific biological mechanism.

#### 4.2.3. Group Differences in Pre-Specified Anatomical ROIs

Based on the schizophrenia dysconnectivity literature [21,29,30,31], four anatomical ROIs were pre-specified prior to analysis: the auditory-language network (T7–T8), the frontal executive network (F3–F4), the superior parietal network (P3–P4), and the inferior parietal/sensory gating area (P7–P8). After applying RGA-NCA, statistically significant group differences were found in all four ROIs (Table 2).

To control the FDR across the four pre-specified ROIs, we applied the BH procedure (α=0.05). All four channel pairs remained significant after FDR correction (BH-adjusted *p* = 0.029 for all comparisons; Table 2).

**Figure 7:**
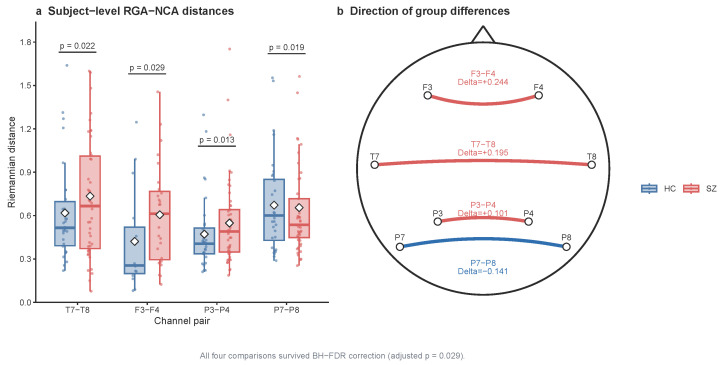
Group-level Riemannian distance differences in the pre-specified ROIs. (**a**) Boxplots and individual subject points show the outlier-screened subject-level RGA-NCA distances used in Table 2. The SZ group showed higher distances for T7–T8, F3–F4, and P3–P4, and a lower distance for P7–P8. Raw *p*-values are shown above each comparison; all four comparisons remained significant after BH-FDR correction (adjusted *p* = 0.029). (**b**) Schematic topological summary of the same group differences. Red connections indicate SZ > HC, and blue indicates SZ < HC.

The group-level topological differences are summarized in Figure 7, which was generated from the same subject-level distances and outlier-screened samples reported in Table 2.

**Increased Frontotemporal and Superior Parietal Riemannian Distances:**

- Auditory-Language Network (T7–T8): The SZ group showed a significant increase in Riemannian distance (*p* = 0.022, FDR-adjusted *p* = 0.029), consistent with greater covariance-structure separation between bilateral temporal channels.
- Frontal Executive Network (F3–F4): A similar increase was observed in the frontal pair (*p* = 0.029, FDR-adjusted *p* = 0.029).
- Superior Parietal Network (P3–P4): The SZ group also showed increased Riemannian distance in this pre-specified parietal pair (*p* = 0.013, FDR-adjusted *p* = 0.029), indicating that the SZ > HC pattern was not restricted to frontotemporal connections.

**Reduced Inferior Parietal Riemannian Distance:**

- Inferior Parietal Lobule/Sensory Gating Area (P7–P8): The SZ group showed a significantly *reduced* Riemannian distance in this region (*p* = 0.019, FDR-adjusted *p* = 0.029). This pattern may be consistent with a lower-complexity phase-locking-like state [4,29,31].

### 4.3. Multi-Dimensional Methodological Validation

#### 4.3.1. Frequency Band Effect and Benchmark Comparison

**Figure 8:**
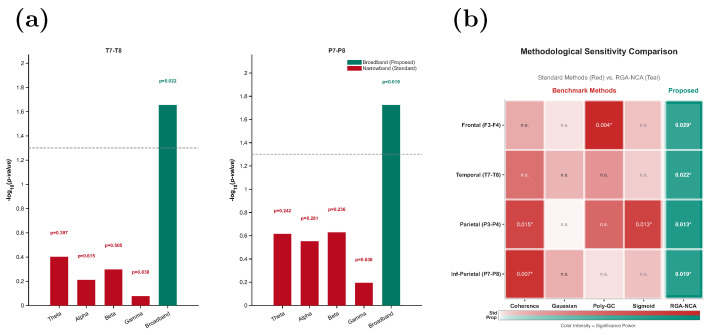
RGA-NCA methodological comparison. (**a**) Frequency-band analysis shows that only broadband analysis reached significance (*p* < 0.05), suggesting that informative group differences may be distributed across frequencies and cross-frequency interactions. (**b**) The methodological sensitivity comparison matrix shows that, in this dataset, RGA-NCA reached statistical significance on all examined core channels (F3–F4, T7–T8, P3–P4, P7–P8), whereas the tested linear coherence and single-kernel methods did not do so consistently. Asterisks denote statistical significance (* *p* < 0.05).

As shown in Figure 8a and summarized in Table 3, RGA-NCA detected statistically significant group differences only when full-band information (1–45 Hz) was retained; the linear coherence baseline was non-significant for F3–F4 and T7–T8. This pattern is consistent with connectivity differences being distributed across a broad frequency range in the analyzed schizophrenia EEG cohort.

#### 4.3.2. Empirical Validation: Contribution of the AAAR Strategy

**Figure 9:**
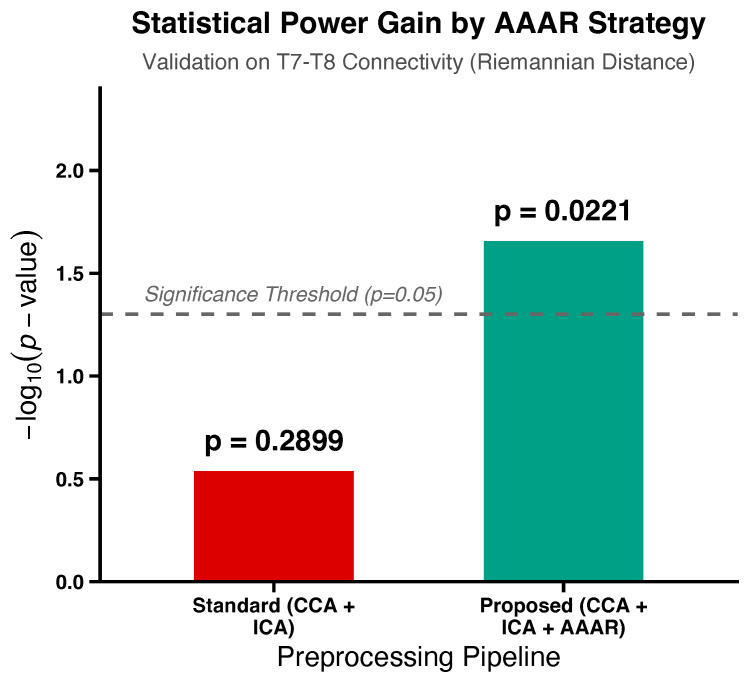
Illustrative gain of the AAAR strategy in the T7–T8 comparison. Bars show $-\log_{10}\left(p\right)$ for the T7–T8 group comparison before and after AAAR. The dashed horizontal line marks the nominal significance threshold (*p* = 0.05). The standard preprocessing result remained below this threshold (*p* = 0.2899), whereas the AAAR result crossed it (*p* = 0.0221).

After introducing the AAAR strategy, the group contrast in the core auditory network (T7–T8) changed substantially. The nominal *p*-value decreased from 0.2899 under the standard preprocessing pipeline to 0.0221 after AAAR, as shown in Figure 9. This result is consistent with the possibility that standard preprocessing pipelines under-regularize frontotemporal signals in this dataset. Since T7–T8 channels are adjacent to facial muscles, their intrinsic neurodynamical connections may be masked by high-amplitude EMG artifacts, causing covariance estimates to become more dispersed. The AAAR strategy, by implementing stricter threshold truncation in these high-noise regions, appears to reduce geometric distortion and increase the visibility of long-range disconnection-like patterns that were less apparent before adaptive preprocessing.

#### 4.3.3. Exploratory Individual-Level Classification

As a secondary analysis, we evaluated whether the RGA-NCA features supported individual-level SZ/HC classification. A linear support vector machine (SVM) classifier [15] trained on the Riemannian tangent space features achieved only about 60% accuracy under leave-one-subject-out cross-validation. This modest accuracy indicates that the present results should be interpreted as group-level connectivity findings rather than as evidence for a clinically useful individual diagnostic classifier.

## 5. Discussion

This study addresses two practical obstacles in EEG network analysis: geometric mismatch and anatomical SNR heterogeneity. The results suggest that combining geometry-aware covariance processing with anatomically adaptive preprocessing can reduce noise sensitivity and increase contrast in group-level schizophrenia analyses. The observed coexistence of local hyper-synchronization-like and long-range desynchronization-like patterns is qualitatively reminiscent of chimera-like organization in coupled systems [32]; however, this analogy is intended as an interpretive heuristic rather than a direct mechanistic identification. The remainder of this discussion therefore focuses on what the framework contributes methodologically, and on what should (and should not) be inferred from the current dataset.

**Figure 10:**
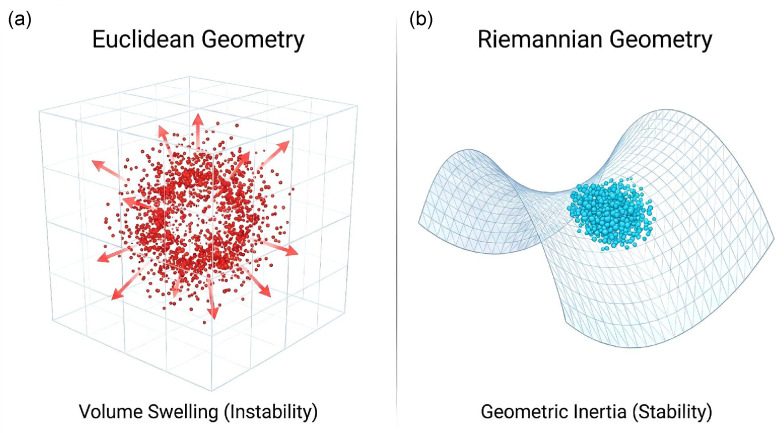
Conceptual schematic of Riemannian geometry under noise perturbation. (**a**) **Euclidean geometry.** In the flat vector space (left), additive noise causes the data distribution (red points) to expand outward along the instability arrows, leading to volume inflation. (**b**) **Riemannian geometry.** On the curved manifold (right, saddle surface), the intrinsic geodesic metric imposes a geometric constraint. The data cluster (cyan points) remains comparatively compact and follows the surface structure, illustrating the proposed noise-resistance interpretation associated with the Riemannian representation.

To make the intuition more concrete, we include the conceptual noise perturbation schematic in Figure 10. We emphasize that it is an explanatory model, not a direct physical proof.

### 5.1. Mitigating Noise-Related Covariance Inflation: From Euclidean Distance to Geodesic Metrics

A central limitation of traditional kernel pipelines in this setting is that they implicitly treat SPD covariance matrices as vectors in a flat Euclidean space. Our theoretical analysis and simulations indicate that violating this geometric assumption can increase noise-related covariance inflation. In the tested settings, Euclidean summaries became more sensitive to increasing noise intensity, which can cause spurious fluctuations from non-stationary noise to be over-interpreted as system-state changes.

RGA-NCA mitigates this geometric distortion by combining AIRM and TSM [9,33]. In our benchmarks, the Riemannian metric showed lower sensitivity to noise-driven volume changes than Euclidean metrics under the tested perturbation settings. This geometry-aware behavior can increase the visibility of structured connectivity differences while preserving nonlinear phase relationships in the analyzed signals. We therefore interpret the larger group-level frontotemporal effects observed with RGA-NCA as evidence that geometry-aware processing may reduce noise-related dispersion relative to the tested Euclidean and linear baselines, not as proof of a unique physiological mechanism.

### 5.2. Role of Hybrid Kernels: Balancing Local Topology and Global Trends

In nonlinear dynamical reconstruction, the kernel choice effectively shapes the feature space. Our parameter sensitivity analysis revealed that model performance follows an inverted “U” shape with the mixing ratio λ, suggesting limitations of single-kernel functions in this dataset. While the Gaussian kernel (RBF) has a smoothing effect on local noise, its rapid decay property can reduce sensitivity to long-range manifold structural information; conversely, the polynomial kernel can capture global high-order interactions but is more sensitive to high-amplitude outliers [17,18].

In our experiments, a hybrid kernel setting in the tested range (λ∈[0.6,0.7]) provided an empirical trade-off between local smoothing (RBF-dominated behavior) and sensitivity to broader interaction structure (polynomial contribution). This multi-scale encoding is therefore interpreted as an auxiliary similarity layer for nonlinear coupling characterization and kernel-mixture sensitivity analysis, whereas the primary ROI-level group contrasts are based on the AIRM distance statistic. More generally, these results support further evaluation of cross-scale feature integration in nonlinear neurophysiological signal analysis, while the exact optimal kernel mixture may vary across datasets and tasks.

### 5.3. Limitations and Future Perspectives

Although RGA-NCA showed greater group contrast than the tested baselines in these experiments, tangent space mapping is still a local linearization approximation. When the brain network state undergoes large distributional shifts, causing the data point distribution to exceed the effective linear range of the tangent space, the accuracy of this method may decrease. In addition, the controlled dynamical-system stress test used covariance matrices derived from delay-embedded scalar trajectories; it should therefore be interpreted as an SPD-manifold stress test under known nonlinear dynamics, not as a generative simulation of multichannel EEG covariance. To address this distinction, Supplementary Simulation S1 adds a 64-channel EEG-like SCM simulation using $C=\left(X-\bar{X}\right){\left(X-\bar{X}\right)}^{⊤}/\left(T-1\right)$, but it remains a stylized consistency check rather than a disease-generative model. Future work will explore using Poincaré disk or other hyperbolic embedding approaches to directly process larger-amplitude manifold nonlinearities without linearization [34]. Furthermore, current anatomical priors are mainly based on group-level statistical assumptions; future work could combine individualized MRI data to construct more individualized subject-specific manifold models [35].

Moreover, despite statistically significant group-level differences, the exploratory individual-level SVM diagnostic accuracy was only about 60%, likely reflecting substantial disease heterogeneity and overlap between groups. Future work could explore deep-learning fusion models (e.g., SPDNet) and multimodal integration (fMRI/DTI) to assess whether individualized prediction can be improved [29,36,37]. The education-level difference between groups (Table 1) is also a potential confound that future studies with larger and better-matched cohorts should address.

## 6. Conclusions

In summary, we present a Riemannian Geometry-based Adaptive Nonlinear Coupling Analysis (RGA-NCA) framework for covariance-based EEG network analysis in schizophrenia. The main methodological contribution is a geometry-aware pipeline that combines AIRM-based manifold distances, tangent space representation, an auxiliary hybrid kernel similarity descriptor, and anatomically adaptive artifact rejection to address noise sensitivity and regional SNR heterogeneity. In benchmark systems and in the analyzed public EEG cohort, the framework showed reduced sensitivity to noise-related covariance inflation and clearer contrast for four pre-specified group-level channel-pair comparisons relative to the tested baselines, all of which remained significant after Benjamini–Hochberg FDR correction. In the noise benchmark, the Riemannian structural sensitivity stayed within 0.936–0.964 across the tested SNR range, whereas the Euclidean baseline fell to 0.000 at −20 dB. The observed connectivity pattern is consistent with coexisting frontotemporal hypo-connectivity-like effects and localized inferior parietal hyper-synchronization-like effects in schizophrenia at the group level. Overall, these findings support geometry-aware covariance modeling as a candidate analysis strategy. At the same time, mechanistic and clinical claims should remain cautious until they are tested in larger, independent, and multimodal datasets.

## Figures and Tables

**Table 1:** Subject demographics and clinical information.

Characteristics	HC Group	SZ Group	Stat.	*p*-Value
Sex (M/F)	26/6	41/8	–	1.000
Age (Years)	38.4±13.9	40.0±13.5	−0.53	0.598
Education (Years)	15.9±2.0	13.6±2.0	5.33	<**0.001**

Age and education were assessed using Welch independent-samples *t*-tests; sex distribution was assessed using Fisher’s exact test. Bold indicates *p* < 0.05.

**Table 2:** Statistical details of Riemannian geometric distances in pre-specified networks. BH-FDR correction applied across the four pre-specified ROIs (α=0.05).

ROIs	Channel Pair	HC Mean	SZ Mean	Raw *p*	FDR *p*
Temporal	T7–T8	0.505	0.700	**0.022**	**0.029**
Frontal	F3–F4	0.362	0.606	**0.029**	**0.029**
Sup. Parietal	P3–P4	0.389	0.490	**0.013**	**0.029**
Inf. Parietal	P7–P8	0.672	0.531	**0.019**	**0.029**

FDR: Benjamini–Hochberg false discovery rate correction; HC: healthy controls; SZ: schizophrenia. Comparisons used two-sided Student two-sample *t*-tests after AAAR-based segment screening and ROI-wise robust outlier screening with MATLAB rmoutliers. Retained $n_{\mathrm{HC}}/n_{\mathrm{SZ}}$ were 26/45 (T7–T8), 14/27 (F3–F4), 28/46 (P3–P4), and 32/42 (P7–P8), with the smaller F3–F4 sample reflecting the strict frontal threshold (SD=2.5). Supplementary Materials report subject-level distances and Welch sensitivity checks. Identical FDR-adjusted *p*-values reflect BH monotonicity across the four pre-specified tests. Bold indicates *p* < 0.05.

**Table 3:** Benchmark comparison: RGA-NCA vs. a linear coherence baseline.

Channel Pair	Linear Baseline	RGA-NCA
F3–F4	NS (*p* = 0.42)	**Sig (*p* = 0.029)**
T7–T8	NS (*p* = 0.08)	**Sig (*p* = 0.022)**
P3–P4	Sig (*p* = 0.01)	**Sig (*p* = 0.013)**
P7–P8	Sig (*p* = 0.01)	**Sig (*p* = 0.019)**

NS: not significant; Sig: significant (*p* < 0.05); raw *p*-values shown. The linear baseline refers to magnitude-squared coherence. All RGA-NCA results remained significant after BH-FDR correction (adjusted *p* = 0.029). Euclidean single-kernel baselines are summarized visually in Figure 8b. Bold indicates significant RGA-NCA results (*p* < 0.05).

## Data Availability

The raw EEG data that support the findings of this study are openly available in Kaggle at https://www.kaggle.com/datasets/broach/button-tone-sz (accessed on 4 March 2026), as a public button-tone SZ/HC EEG dataset associated with the auditory feedback paradigm described by Ford et al. Derived subject-level feature tables and cleaned analysis scripts supporting the reported group-level statistics are provided in the Supplementary Materials.

## Supplementary Materials

The following supporting information can be downloaded at https://www.mdpi.com/article/10.3390/e28060644/s1.
