# Supplementary material for: Riemannian Geometry for Noise-Robust Covariance Network Analysis of Schizophrenia EEG: Geometric-Entropic Signatures of Dysconnectivity
Source: Entropy (Basel). 2026 Jun 8;28(6):644. doi: 10.3390/e28060644 (PMC13297899; doi:10.3390/e28060644)
Supplement: Supplementary file 1 [file entropy-28-00644-s001.zip › Supplementary_Materials/Supplementary_Materials_entropy-4356966.pdf]

Supplementary Materials

Riemannian Geometry for Noise-Robust Covariance Network Analysis of Schizophrenia EEG: Geometric-Entropic Signatures of Dysconnectivity

Rui Song, Jinhan He and Jun Wang

This document collects the supplementary data tables (Tables S1–S6), the supplementary multichannel EEG-like simulation (Simulation S1, Figure S1, Table S7), and the cleaned analysis code (Code S1–S4). The full numeric tables are additionally provided as the accompanying CSV files listed under Supplementary Files; compact versions are typeset below to support the statements made in the main text.

Supplementary Data Tables (Empirical Analysis)

Table S1 summarizes the structure of the subject-level table. The complete table (510 subject × channel-pair rows) is provided as `rga_nca_subject_level_distances.csv`.

Table S1: Subject-level RGA-NCA distance table (column dictionary). The full per-subject, per-channel-pair values are provided in the accompanying CSV file.

Column	Description
`subject`, `group`	Subject identifier and group label (HC or SZ)
`channel_pair`, `roi_label`	Bilateral channel pair and its anatomical ROI label
`coupling_score`	Hybrid-kernel tangent-space similarity descriptor
`riemann_distance`	Subject-level AIRM (Riemannian) distance statistic
`primary_roi`	Whether the pair is one of the four pre-specified ROIs
`retained_by_mad_rule`	Retained (True) or removed by MATLAB `rmoutliers`
`mad_center/lower/upper`	Median/MAD screening center and bounds

Table S2 reports retained sample sizes after robust outlier screening, group means, the primary two-sample Student t-test, the Welch sensitivity check, and the BH-FDR adjusted p-values. These values correspond to Table 2 of the main text. The full table is provided as `rga_nca_roi_statistics.csv`.

Table S2: Primary ROI group statistics with Welch sensitivity checks. n shown as raw→retained after `rmoutliers` screening; means as mean ± SD. Student p is the primary test; Welch p is the sensitivity check; BH-FDR is across the four pre-specified ROIs.

ROI (pair)	n_{HC}	n_{SZ}	HC mean	SZ mean	Student p / Welch p	BH-FDR p
Auditory-language (T7–T8)	30 → 26	46 → 45	0.505 ± 0.178	0.700 ± 0.401	0.0221 / 0.0065	0.029
Frontal executive (F3–F4)	15 → 14	27 → 27	0.362 ± 0.283	0.606 ± 0.345	0.0288 / 0.0215	0.029
Superior parietal (P3–P4)	32 → 28	49 → 46	0.389 ± 0.119	0.490 ± 0.189	0.0133 / 0.0061	0.029
Inferior parietal (P7–P8)	32 → 32	49 → 42	0.672 ± 0.330	0.531 ± 0.165	0.0189 / 0.0323	0.029

Table S3 reports the FDR-corrected directional Granger group comparisons; no channel pair reached significance in either direction. The full feature table and test output are provided as `directional_granger_features.csv` and `directional_granger_group_tests_fdr.csv`.

Table S3: Directional Granger baseline group comparisons (BH-FDR corrected). Raw and FDR-adjusted p-values for left→right (L2R) and right→left (R2L) directions; none significant (ns).

Channel	L2R p	L2R p_{FDR}	R2L p	R2L p_{FDR}
Fp1–Fp2	0.540	0.720	0.699	0.941
F3–F4	0.911	0.990	0.914	0.941
C3–C4	0.393	0.720	0.821	0.941
P3–P4	0.990	0.990	0.941	0.941
O1–O2	0.348	0.720	0.256	0.941
F7–F8	0.472	0.720	0.920	0.941
T7–T8	0.512	0.720	0.690	0.941
P7–P8	0.457	0.720	0.510	0.941

Table S4 reports left→right raw p-values for the Gaussian, polynomial, and sigmoid kernels at the four pre-specified ROI pairs. No single Euclidean kernel produced a consistent significant ROI pattern after correction. The full table (both directions, t-values, and Cohen's d for all eight pairs) is provided as `euclidean_kernel_baseline_group_tests.csv`.

Table S4: Euclidean single-kernel baseline at the pre-specified ROIs. Left→right raw p-values; none survive FDR correction across kernels and ROIs.

ROI (pair)	Gaussian p	Polynomial p	Sigmoid p
F3–F4	0.911	0.004	0.628
T7–T8	0.512	0.253	0.742
P3–P4	0.990	0.060	0.886
P7–P8	0.457	0.888	0.619

Table S5 compares the magnitude-squared coherence baseline with RGA-NCA at the four pre-specified ROIs (raw p-values), matching Table 3 of the main text. Provided as `linear_coherence_vs_rga_summary.csv`.

Table S5: Linear coherence baseline versus RGA-NCA. Raw p-values for the four pre-specified ROI pairs.

Channel pair	Linear coherence p	RGA-NCA p
F3–F4	0.42	0.029
T7–T8	0.08	0.022
P3–P4	0.01	0.013
P7–P8	0.01	0.019

Table S6 reports the T7–T8 group-comparison p-value before and after AAAR, used in the statistical-power illustration (Figure 9 of the main text). Provided as `aaar_t7t8_power_summary.csv`.

Table S6: AAAR ablation (T7–T8 connectivity). Group-comparison p-value and $-\log_{10} p$ before and after adding the AAAR step.

Preprocessing condition	Raw p	$-\log_{10} p$
Standard preprocessing only	0.2899	0.538
Standard preprocessing + AAAR	0.0221	1.656

Supplementary Simulation S1. Multichannel EEG-like Channel Covariance Simulation

The controlled dynamical-system analysis in the main manuscript uses covariance matrices derived from delay-embedded scalar trajectories. To provide an additional check with the same matrix form as the empirical EEG analysis, we added a multichannel EEG-like simulation in which sample covariance matrices were computed directly from channel-by-time data,

$$X \in \mathbb{R}^{N_{\text{channels}} \times T}, \qquad C = \frac{(X - \bar{X})(X - \bar{X})^\top}{T - 1}. \tag{1}$$

This supplementary simulation was designed as a methodological consistency check, not as a generative model of schizophrenia EEG.

Simulation Design

We simulated $N_{\text{channels}} = 64$ EEG channels and $T = 500$ samples per segment, matching the empirical RGA-NCA window length. Each Monte Carlo repetition used the empirical sample sizes of the main analysis ($n_{\text{HC}} = 32$, $n_{\text{SZ}} = 49$). The clean multichannel signal for group $g \in \{\text{HC}, \text{SZ}\}$ was generated as

$$X_g = L_g^\top Z, \tag{2}$$

where L_g is the upper Cholesky factor of the group covariance template, satisfying $L_g^\top L_g = \Sigma_g$, and Z is a temporally colored source matrix. The temporal driver used autoregressive colored noise with region-specific oscillatory components: occipital alpha-like activity, temporal theta-like activity, and frontal beta-like activity. This construction introduces EEG-like temporal autocorrelation while retaining direct channel-wise covariance estimation.

The HC covariance template was built from spatially smooth scalp correlations plus region-level block structure. The SZ template was created by implanting four predefined channel-wise covariance perturbations into the HC template and then projecting the result back to the SPD cone:

$$\Sigma_{\text{SZ}} = \Pi_{\mathcal{S}_{++}} \left(\Sigma_{\text{HC}} + \sum_{(i,j)\in\mathcal{P}} \delta_{ij} (e_i e_j^\top + e_j e_i^\top) \right). \tag{3}$$

The implanted perturbations were: T7–T8 ($\delta = -0.30$), F3–F4 ($\delta = -0.25$), P3–P4 ($\delta = -0.20$), and P7–P8 ($\delta = +0.24$). These perturbations were used only to create a known ground-truth covariance contrast.

Sensor noise was added as

$$Y = X + E, \tag{4}$$

with heterogeneous channel variance. Noise weights were highest for frontotemporal channels, lowest for occipital channels, and intermediate elsewhere. Signal-to-noise ratios were tested at +20, +10, 0, and −10 dB. For each SNR, 30 Monte Carlo repetitions were run.

For both the Riemannian and Euclidean analyses, SCMs were diagonally loaded and trace-normalized before comparison:

$$\tilde{C} = \frac{C + \epsilon \bar{d} I}{\text{Tr}(C + \epsilon \bar{d} I)}, \qquad \bar{d} = \frac{1}{N_{\text{channels}}} \text{Tr}(C), \quad \epsilon = 0.05. \tag{5}$$

The Riemannian representation used the affine-invariant tangent-space descriptor, whereas the Euclidean baseline used vectorized SCM differences with the Frobenius geometry.

Evaluation Metrics

Three quantities were computed. First, whole-SCM contrast recovery measured whether the noisy HC–SZ difference vector preserved the clean implanted HC–SZ covariance contrast. This was reported as cosine similarity between the clean and noisy contrast vectors. Second, ROI-pair recovery evaluated the four implanted channel pairs directly, using ROI-level cosine similarity and sign accuracy between the clean and noisy ROI contrast vectors. Third, a sensitivity grid repeated the simulation across four implanted-effect scales (0.50×, 0.75×, 1.00×, and 1.25×) and four SNR levels to test whether the AIRM advantage depended on a single selected effect size.

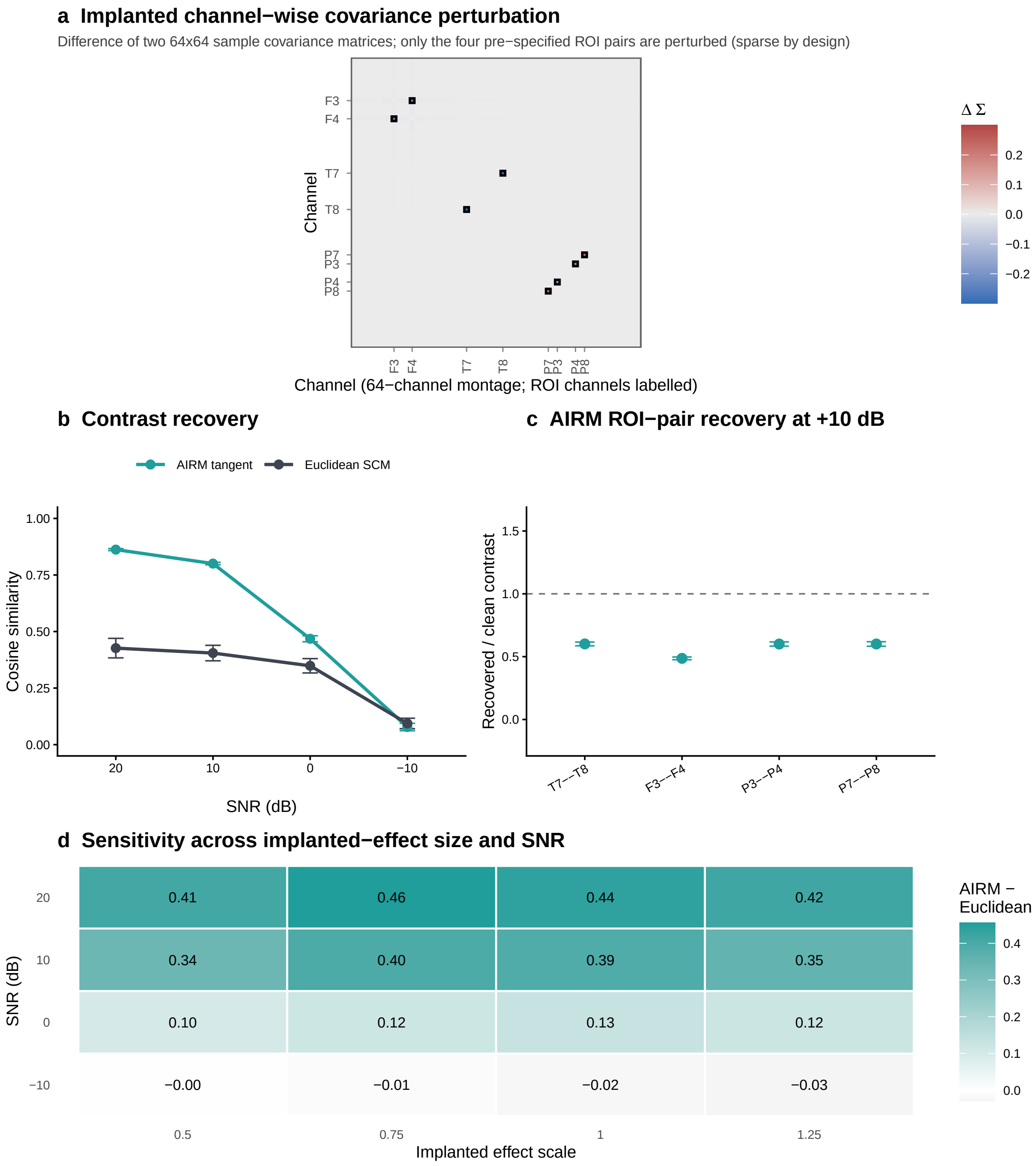

Figure S1: Supplementary multichannel EEG-like channel covariance simulation. (**a**) Implanted channel-wise covariance perturbation $\Delta\Sigma = \Sigma_{\mathrm{SZ}} - \Sigma_{\mathrm{HC}}$ in a 64-channel SCM. The matrix is sparse by design, with perturbations restricted to the four predefined channel pairs. (**b**) Recovery of the implanted clean HC–SZ full-SCM covariance contrast after adding heterogeneous sensor noise. (**c**) AIRM ROI-pair recovery at +10 dB, plotted as recovered contrast divided by the clean AIRM ROI contrast; values above zero indicate preserved direction. (**d**) Parameter-sensitivity grid showing the mean AIRM advantage over the Euclidean SCM baseline in full-SCM contrast recovery across implanted-effect scales and SNR levels. Points and error bars in panels b–c show Monte Carlo mean ± SD across 30 repetitions.

Table S7: Summary of the multichannel EEG-like covariance simulation. Values are Monte Carlo mean ± SD across 30 repetitions.

SNR (dB)	AIRM full-SCM cosine	Euclidean full-SCM cosine	AIRM ROI cosine	Euclidean ROI cosine	AIRM sign accuracy	Euclidean sign accuracy
+20	0.862 ± 0.005	0.427 ± 0.043	0.999 ± 0.001	0.997 ± 0.003	1.00 ± 0.00	1.00 ± 0.00
+10	0.800 ± 0.005	0.405 ± 0.034	0.995 ± 0.001	0.996 ± 0.003	1.00 ± 0.00	1.00 ± 0.00
0	0.468 ± 0.013	0.349 ± 0.032	0.979 ± 0.008	0.994 ± 0.004	1.00 ± 0.00	1.00 ± 0.00
−10	0.078 ± 0.016	0.093 ± 0.023	0.892 ± 0.064	0.907 ± 0.052	1.00 ± 0.00	0.99 ± 0.05

The simulation confirms that the additional covariance benchmark can be formulated using the same channel-wise SCM construction as the empirical EEG analysis. Direct ROI-pair recovery was high in both representations, confirming that the implanted channel-pair perturbations were recoverable in the simulated SCMs. The main advantage of AIRM appeared in whole-SCM contrast recovery: under moderate noise levels (+20 and +10 dB), the AIRM tangent-space representation preserved the global implanted HC–SZ covariance contrast more faithfully than the Euclidean SCM baseline. The parameter-sensitivity grid further showed that the AIRM advantage in whole-SCM contrast recovery was positive across implanted-effect scales from $0.50\times$ to $1.25\times$ at +20, +10, and 0 dB, but vanished under severe noise (−10 dB). Thus, this supplementary analysis supports the consistency of the covariance formulation while preserving the cautious interpretation that it is a stylized methodological check rather than a disease-generative simulation.

Supplementary Code

Code S1. Clean MATLAB implementation of the RGA-NCA analysis pipeline and core coupling/log-map distance function (`run_rga_nca_analysis.m`, `compute_riemann_hybrid_coupling.m`).

Code S2. Clean MATLAB implementation of the linear coherence benchmark and the exploratory leave-one-subject-out SVM analysis (`run_linear_coherence_benchmark.m`, `run_svm_classification.m`).

Code S3. Base-R script reproducing the primary ROI statistics and BH-FDR correction from the supplied CSV files (`reproduce_statistics.R`).

Code S4. R script generating the multichannel EEG-like covariance simulation, Figure S1, and Table S7 (`Code_S4_eeg_like_covariance_simulation.R`).

Supplementary Files

The following files accompany this supplement.

Empirical data tables (Tables S1–S6):

- `rga_nca_subject_level_distances.csv`: Table S1, subject-level RGA-NCA distances.
- `rga_nca_roi_statistics.csv` (and `rga_nca_roi_statistics_recomputed.csv`): Table S2, primary ROI statistics and Welch checks.
- `directional_granger_features.csv`, `directional_granger_group_tests_fdr.csv`: Table S3, directional Granger baseline.
- `euclidean_kernel_baseline_group_tests.csv`: Table S4, Euclidean single-kernel baseline.
- `linear_coherence_vs_rga_summary.csv`: Table S5, linear coherence versus RGA-NCA.
- `aaar_t7t8_power_summary.csv`: Table S6, AAAR ablation.

Simulation S1 files (Figure S1, Table S7):

- `Figure_S1_EEG_like_covariance_simulation.pdf`: vector version of Supplementary Figure S1.
- `Supplementary_Table_S7_EEG_like_summary.csv`: summary statistics for Table S7.
- `Supplementary_EEG_like_monte_carlo_summary.csv`: repetition-level Monte Carlo summary.
- `Supplementary_EEG_like_subject_metrics.csv`: subject-level simulated metrics.
- `Supplementary_EEG_like_roi_pair_recovery.csv`: ROI-pair recovery values for the four implanted channel pairs.
- `Supplementary_EEG_like_parameter_grid.csv` and `Supplementary_EEG_like_parameter_grid_summary.csv`: sensitivity-grid results and summary.

Code (Code S1–S4):

- `run_rga_nca_analysis.m`, `compute_riemann_hybrid_coupling.m`: Code S1.
- `run_linear_coherence_benchmark.m`, `run_svm_classification.m`: Code S2.
- `reproduce_statistics.R`: Code S3.
- `Code_S4_eeg_like_covariance_simulation.R`: Code S4.
