# Supplementary material for: Riemannian Geometry for Noise-Robust Covariance Network Analysis of Schizophrenia EEG: Geometric-Entropic Signatures of Dysconnectivity
Source: Entropy (Basel). 2026 Jun 8;28(6):644. doi: 10.3390/e28060644 (PMC13297899; doi:10.3390/e28060644)
Supplement: Supplementary file 1 [file entropy-28-00644-s001.zip › Supplementary_Materials/data/Figure_S1_EEG_like_covariance_simulation.pdf]

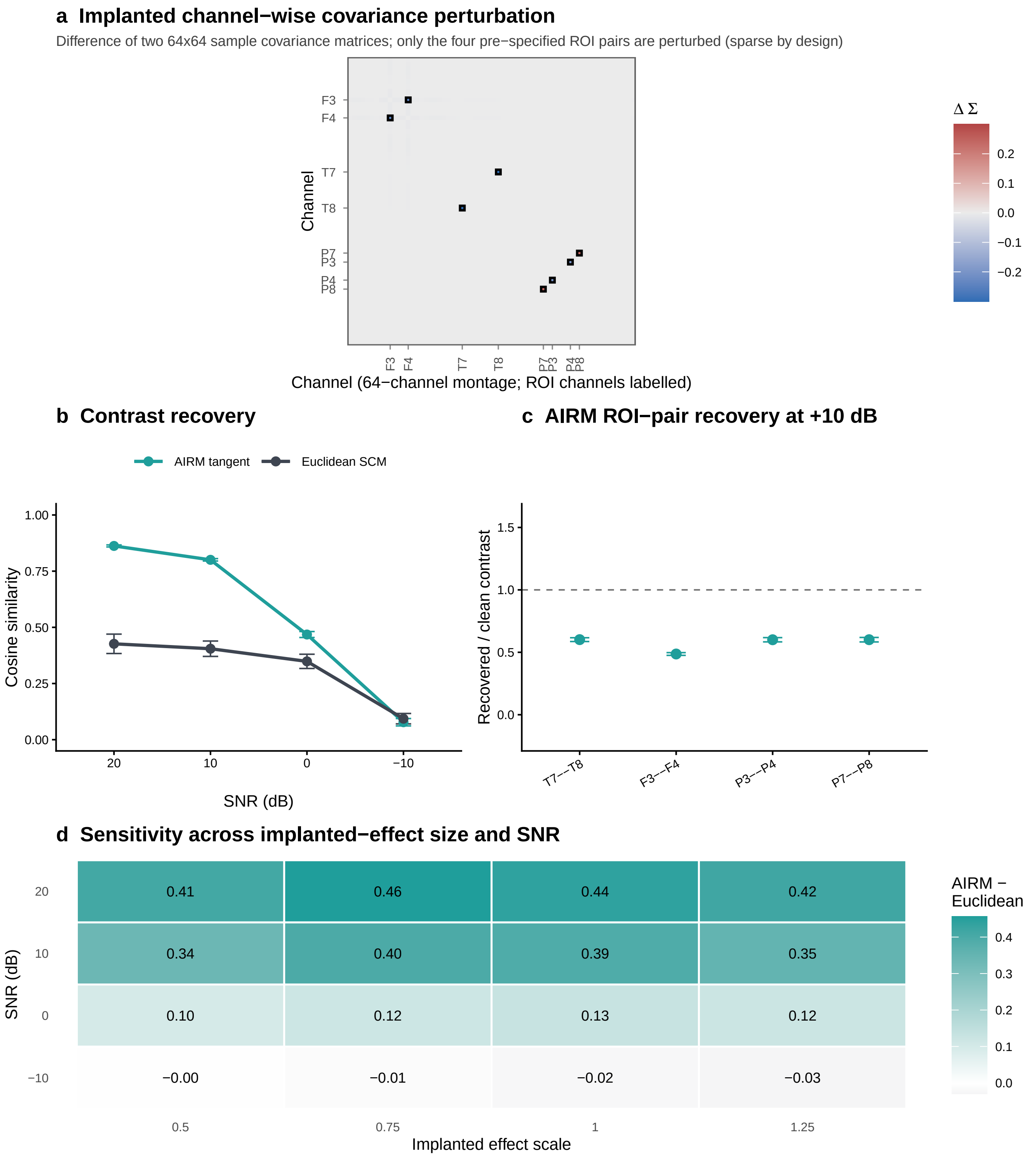
a Implanted channel−wise covariance perturbation
Difference of two 64x64 sample covariance matrices; only the four pre−specified ROI pairs are perturbed (sparse by design)
Channel
F3
F4
T7
T8
P7
P3
P4
P8
Channel (64−channel montage; ROI channels labelled)
Δ Σ
0.2
0.1
0.0
−0.1
−0.2
b Contrast recovery
AIRM tangent
Euclidean SCM
Cosine similarity
1.00
0.75
0.50
0.25
0.00
20
10
0
−10
SNR (dB)
c AIRM ROI−pair recovery at +10 dB
Recovered / clean contrast
1.5
1.0
0.5
0.0
T7−−T8
F3−−F4
P3−−P4
P7−−P8
d Sensitivity across implanted−effect size and SNR
SNR (dB)
20
10
0
−10
0.41
0.46
0.44
0.42
0.34
0.40
0.39
0.35
0.10
0.12
0.13
0.12
−0.00
−0.01
−0.02
−0.03
0.5
0.75
1
1.25
Implanted effect scale
AIRM − Euclidean
0.4
0.3
0.2
0.1
0.0
