# Supplementary figures and images for: Riemannian Geometry for Noise-Robust Covariance Network Analysis of Schizophrenia EEG: Geometric-Entropic Signatures of Dysconnectivity
Source: Entropy (Basel). 2026 Jun 8;28(6):644. doi: 10.3390/e28060644 (PMC13297899; doi:10.3390/e28060644)

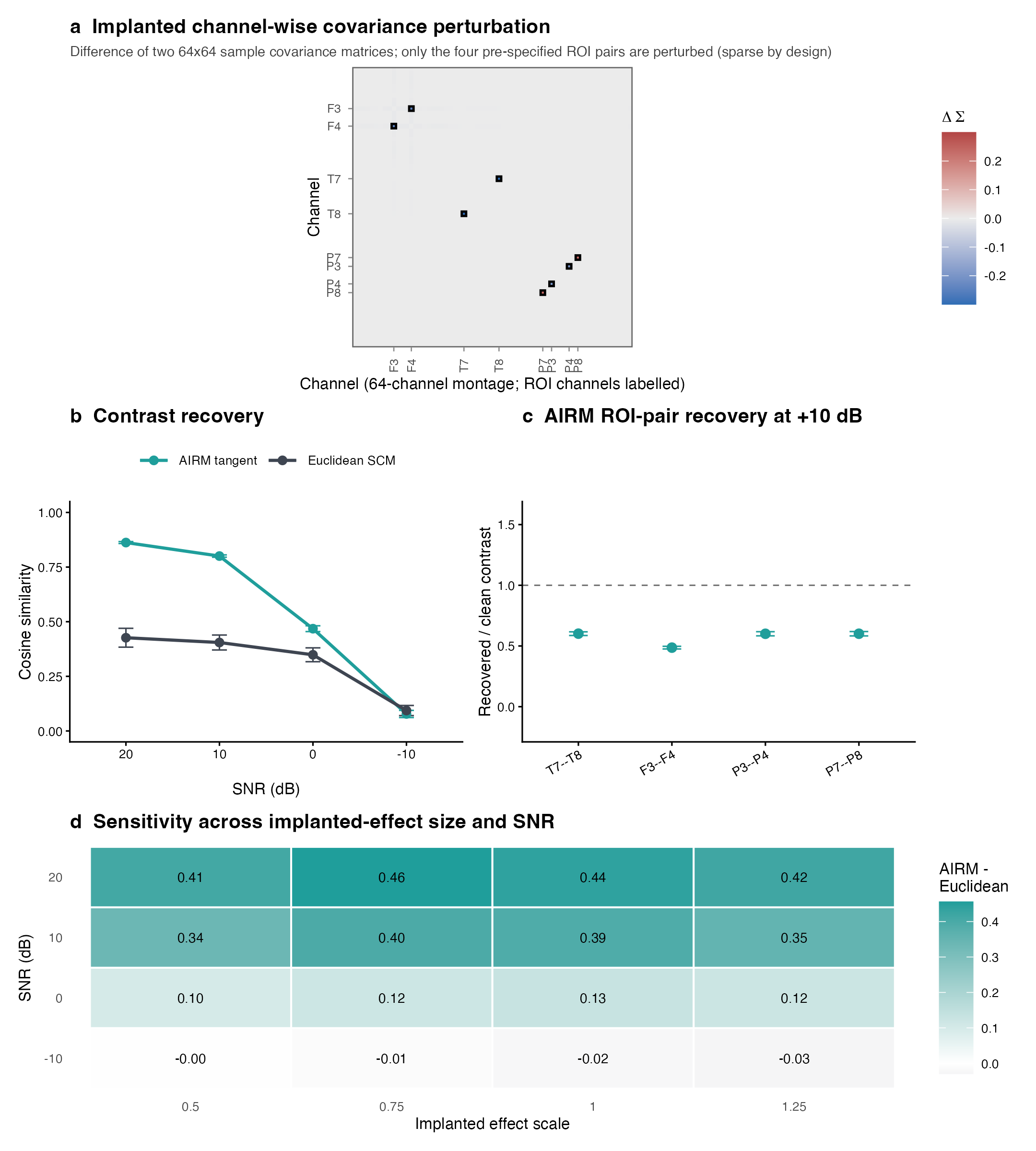

Supplement: Supplementary file 1 [file entropy-28-00644-s001.zip › Supplementary_Materials/data/Figure_S1_EEG_like_covariance_simulation.png]

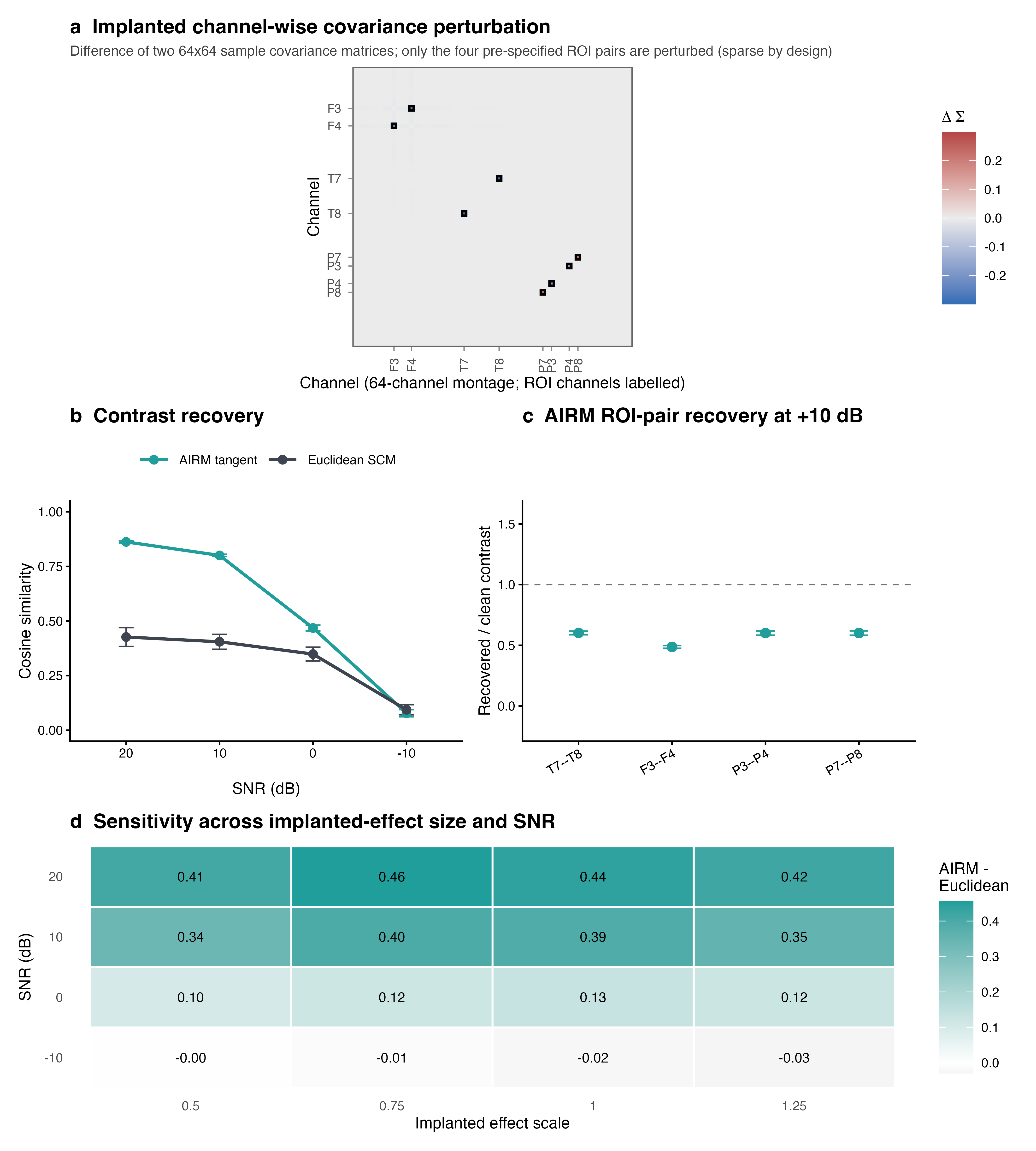

Supplement: Supplementary file 1 [file entropy-28-00644-s001.zip › Supplementary_Materials/data/Figure_S1_EEG_like_covariance_simulation.tiff]
